# N^β^-methylation changes the recognition pattern of aza-β^3^-amino acid containing peptidomimetic substrates by protein kinase A

**DOI:** 10.1186/2191-2858-1-16

**Published:** 2011-11-08

**Authors:** Ksenija Kisseljova, Michèle Baudy-Floc'h, Aleksei Kuznetsov, Jaak Järv

**Affiliations:** 1 Institute of Chemistry, University of Tartu, 14A Ravila Street, 50411 Tartu, Estonia; 2 Groupe 'Ciblage et Auto-Assemblages Fonctionnels', UMR CNRS 6226, Institut de Chimie, Université de Rennes 1, 263 Av. du Général Leclerc, F-35042 Rennes Cedex, France

**Keywords:** Peptidomimetic, *N*^β^-Me-aza-β^3^-amino acid, c-AMP-dependent protein kinase A, peptidomimetics recognition, phosphorylation, protein kinase specificity

## Abstract

The protein kinase A (PKA)-catalyzed phosphorylation of peptide substrate RRASVA analogs, containing *N*^β^-Me-aza-β^3^-amino acid residues in all subsequent positions, was studied. This work follows along the lines of our previous research of the phosphorylation of aza-β^3^-analogs of RRASVA (the shortest active substrate of PKA) and allows characterizing the influence of N^β^-methylation of aza-β^3^-amino acid residues on substrate recognition by PKA on substrate binding and phosphorylation steps. It was found that the effect of N^β^-methylation was dependent upon the position of the structure alteration. Moreover, the presence of a single N^β^-methylation site in the substrate changed the recognition pattern of this series of peptidomimetics, strongly affecting the phosphorylation step. Structure modeling of aza-β^3^- and *N*^β^-Me-aza-β^3^-containing substrates revealed that N^β^-methylation of aza-β^3^-moieties changed the peptide bond geometry from *trans*- to *cis*-configuration in -CO-NMe- fragments, with an exception for the *N*-terminally methylated *N*^β^-Me-aza-β^3^-RRRASVA (with the *N*-terminal amino group not participating in the peptide bond) and RRAS-*N*^β^-Me-aza-β^3^-VA. As has been shown in literature, this conformational preference of the backbone has a significant influence on the flexibility of the peptide substrate chain. Following our results, this property seems to have significant influence on the recognition of the amino acid side groups by the enzyme binding site, and in the case of PKA this structural modification was decisive for the phosphate transfer step of the catalytic process.

## Background

*N*-Methylation is one of the most common ways of peptide backbone modification [1]. Replacement of the amide group hydrogen atom by a bulk methyl group results in disruption of backbone hydrogen bonding, restricts the conformation of the side chains [2], increases hydrophobicity by reducing the number of possible intramolecular hydrogen bonds [3], and decreases peptide bond preference for *trans*-configuration [3,4]. All these changes have made this way of backbone modification an attractive tool of peptidomimetic design.

**Figure 1:**
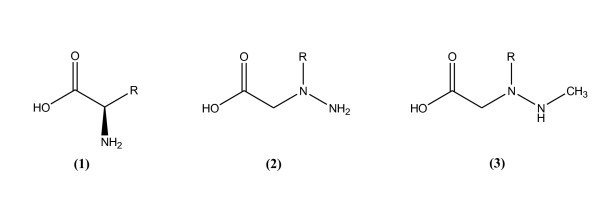
**l-amino acid (1), its aza-β^3^-analog (2) and *N*^β^-methyl-aza-β^3^-analog (3)**.

In our previous article [5] we have studied the effect of aza-β^3^-amino acids (Figure 1, center) on the kinetics of peptidomimetic substrates phosphorylation by the protein kinase A (PKA). These substrates mimicked the well-known minimum substrate RRASVA of PKA [6], leaving the phosphorylatable serine residue unchanged and conserving the sequence of the amino acid side-chains. As the reaction step includes the transfer of the γ-phosphate group of ATP to the serine -OH group of the substrate, the reacting groups must closely be positioned in the enzyme active site in the course of the preceding binding step [7]. Therefore, the phosphorylation reaction along with the binding step of peptide or peptidomimetic molecules should adequately represent the pattern of molecular recognition of these substrates in the active site of the enzyme, and differences in reactivity should characterize differences in this recognition mechanism. All the peptidomimetics described were phosphorylated by PKA.

In this study, the influence of N^β^-methylation of the same series of peptidomimetic substrates on their recognition in the enzyme binding site was investigated. Therefore, peptidomimetic RRASVA analogs with *N*^β^-methyl-aza-β^3^-mutations in five different positions were prepared and their phosphorylation by PKA was studied. It was found that N^β^-methylation significantly affects the substrate recognition pattern and the effects observed were dependent upon *N*^β^-aza-β^3^-substitution position.

## Results and discussion

The list of *N*^β^-methylated peptidomimetics is given in Table 1. These compounds were synthesized by the common SPPS methodology, using the corresponding *N*^β^-methyl-aza-β^3^-amino acids, the preparation of which was reported previously [8]. *N*^β^-methyl-aza-β^3^-amino acids were coupled as reported previously [5], using TBTU/HOBT as activators. For the coupling of amino acid following the *N*^β^-methyl-aza-β^3^-residue, stronger activation was required and HATU/HOBT was used [9].

**Table 1:** Phosphorylation of *N*^β^-methylated peptidomimetic substrates by PKA catalytic subunit at ATP concentration 100 μM, 30°C, 50 mM TRIS/HCl, pH 7

	Substrate	*K*_m _(μM)	10^2 ^*k*_cat _(μmole mg^-1 ^s^-1^)	10^2 ^*k*_II _(L mg^-1 ^s^-1^)
I	**R**RASVA	209 ± 24	24.4 ± 1.5	0.098 ± 0.010
II	R**R**ASVA	79 ± 18	11.9 ± 1.0	0.11 ± 0.07
III	RR**A**SVA	91 ± 15	5.5 ± 0.3	0.034 ± 0.005
IV	RRAS**V**A	118 ± 36	16 ± 2.0	0.12 ± 0.02
**V**	RRASV**A**	57 ± 17	5.0 ± 0.4	0.090 ± 0.015
	RRASVA (from [16])	11.1 ± 3.5	36 ± 3	3.2 ± 0.1

**AA**, *N*^β^-Me-aza-β^3^-amino acid.

It was found that all the synthesized *N*^β^-methylated aza-β^3^-peptides were phosphorylated by PKA and the results of the kinetic study of their phosphorylation are listed in Table 1. The phosphorylation reactions followed the classical Michaelis-Menten rate equation and therefore all substrates were characterized by the *K*_m _and *k*_cat _values, which correspond to the constant ATP concentration of 0.1 mM. The initial linear part of the Mihaelis-Menten plot was used for the calculation of the second order rate constants *k*_II _as described in [10] and these values are also listed in Table 1. Agreement between the parameters *k*_II _and values of the ratio of *k*_cat_/*K*_m _for substrates confirms the applicability of the Michelis-Menten rate equation for the description of the kinetic data [11].

**Figure 2:**
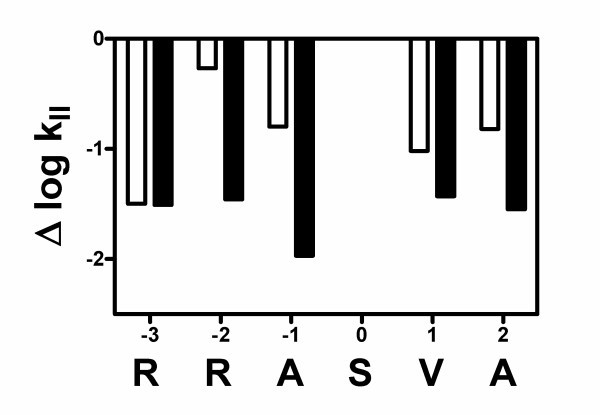
**The effect of the substitution of amino acids in peptide RRASVA by *N*^β^-Me-aza-β^3^-amino acids (black bars) and aza-β^3^-amino-acids (white bars) on the phosphorylation of peptidomimetic substrates by PKA**.

It can be seen in Table 1 that the presence of the *N*^β^-methyl-aza-β^3^-moiety significantly reduced the reactivity of all the peptidomimetics in comparison to the parent peptide RRASVA. It was also noteworthy that this effect was strongly depending upon the location of the amino acid analog in substrate sequence. Although the substrates with non-methylated aza-β^3^-amino acids mutations demonstrated similar behavior in general, it can be seen from Figure 2 that in the case of N^β^-methylation the effects became more significant, if assessed on the basis of the second-order phosphorylation constants.

The Δlog *k*_II _values shown in this figure represent the logarithm of the ratio of the *k*_II _values for peptidomimetics and the parent peptide RRASVA, and also include data from our previous article and are represented by white bars [5]. Second, it should be noted that in this numbering system (Figure 2) the position of the phosphorylatable serine is denoted as zero, amino acid residues to the left and right of it--with negative and positive numerals, respectively.

It is noteworthy that the reactivities of substrates *N*^β^-Me-aza-β^3^-RRASVA and aza-β^3^-RRASVA were practically similar, indicating that methylation of the N-teminal amino group of these compounds had no effect on their recognition by the enzyme. Not surprisingly, the *K*_m _and *k*_cat _values of these two substrates were also similar. However, apart from the N-terminal position N^β^-methylation of aza-β^3^-moiety caused significant differences in reactivity of the methylated and non-methylated compounds, whereby the latter substrates were always more efficiently phosphorylated (Figure 2). In one case, if the structure of aza-β^3^-alanine residue in position -1 was methylated, the effect reached almost two orders of magnitude.

**Figure 3:**
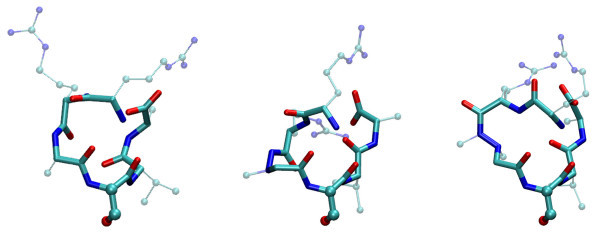
**Comparison of the backbone structure of computer-modeled conformations of RRASVA (left) RR-aza-β^3^-ASVA (center), and RR-*N*^β^-Me-aza-β^3^-ASVA (right)**. The backbones are shown as stick models, while three atoms of the serine residues, which were similarly fixed in all three molecules, were shown as bold ball-and-stick models. The side groups of both arginine residues were also shown by ball-and-stick models and their different orientation can be observed.

This significant decrease of reactivity can be explained by the fact that N^β^-methylation favored *cis*-configuration of -CO-NMe- group. This tendency of peptide bond configuration shifting from *trans *to *cis *has extensively been discussed in multiple articles (to name but a few, [3,12-15]). In the present case this tendency was also observed in the case of results of computer modeling of these peptidomimetics, and was illustrated by the calculated optimal structure of RR-*N*^β^-Me-aza-β^3^-ASVA in Figure 3, while in RR-aza-β^3^-ASVA the corresponding non-methylated moiety has *trans*-geometry (compare with Figure 3 structures on the right and in the center).

For several other positions, the decrease in reactivity was not so significant; however, the *N*^β^-methylated peptidomimetics were phosphorylated at about 30 times lower rate when compared to RRASVA. In *N*^β^-Me-aza-β^3^-RRASVA, the *trans*-geometry of the -CO-NMe- bond was observed in computer models, which was not the surprising result as the N-terminal nitrogen atom was methylated. Indeed, reactivity of this substrate differed much less from its non-methylated counterpart aza-β^3^-RRASVA. Therefore, the amide group *cis- *and *trans-*configurations seem to play a crucial role in the determination of substrate reactivity. Structure calculations have also shown *trans*-configuration of -CO-NMe- bond for RRAS-*N*^β^-Me-aza-β^3^-VA analog. It can be observed in Figure 2 that the difference in reactivity of this substrate (in terms of Δlog *k*_II_) is less than that of between other members of the reaction series and their non-methylated aza-β^3^-counterparts. The compound (*N*^β^-Me-aza-β^3^-RRRASVA) was an understandable exception, as the N-terminal amino group does not participate in peptide bond formation.

**Figure 4:**
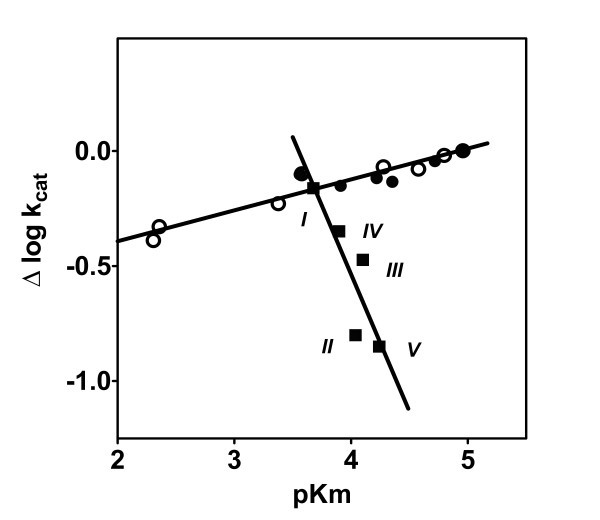
**Interrelationships between log *k*_cat _and *pK*_m _values in the PKA catalyzed phosphorylation reaction of *N*^β^-Me-aza-β^3^-amino acid containing peptidomimetics**. ('black square', numbers correspond to Table 1), aza-β^3^amino acid containing peptidomimetics ('black circle', data from our previous report [5]) and peptide substrates ('white circle', data from [16] for the following peptides: LRRASLG; RRASLG; LRRASLG; LRKASLG LARASLG; RASLG; LHRASLG and RRASVA [16]).

The effects observed in log *k*_II _values should certainly summarize the influence of acting specificity factors upon the *pK*_m _and log *k*_cat _values. It can be seen in Table 1 that these two kinetic parameters changed in a rather similar way within the series of *N*^β^-Me-aza-β^3^-derivatives. On the other hand, reactivity of non-methylated aza-β^3^-derivatives was governed mostly by variations in binding effectiveness, characterized by the *pK*_m _values, as was shown in our previous report [5]. This situation was similar to the PKA specificity pattern for peptide substrates, where variation in the peptide length and, more importantly, the sequence of amino acids only has a minor effect upon the *k*_cat _values, while the different recognition of substrates was mostly governed by their binding step [16]. This situation was clearly illustrated by the log *k*_cat _versus *pK*_m _plot for aza-β^3^-derivatives and common peptides, as shown in Figure 4.

For simplification, the *k*_cat _value for RRASVA phosphorylation was used for the normalization of kinetic data and the calculation of the Δlog *k*_cat _values as shown in Figure 4. However, N^β^-methylation of the aza-β^3^-group essentially changed this regularity, as the Δlog *k*_cat _values for these compounds varied significantly within the reaction series.

It is noteworthy that the Δlog *k*_cat _versus *pK*_m _plot for *N*^β^-methylated peptidomimetics had a negative slope and the phosphorylation rate of these compounds decreased if the *pK*_m _values increased (Figure 4). This change in the specificity pattern of *N*^β^-methyl-aza-β^3^-derivatives, when compared to that of aza-β^3^-derivatives and common peptides, was surprising, as structures of these peptidomimetics were not very different from each other, and changes in substrate backbone occurred at a distance from the phosphorylatable serine residue. On the other hand, this change in specificity pattern confirmed the significant role of peptide or peptidomimetic backbone flexibility that seems to be a crucial factor for matching ligand side-chains with its binding sites. N^β^-methylation obviously limits this kind of flexibility [2].

## Conclusions

The comparison of kinetic data of the PKA catalyzed phosphorylation of RRASVA analogs with *N*^β^-Me-aza-β^3^- and aza-β^3^-mutations of all subsequent positions revealed that N^β^-methylation changed the pattern of substrate recognition by this enzyme. This change manifested itself in the different relationships between the binding effectiveness of substrates and the catalytic activity of the enzyme, characterized in terms of the log *k*_cat _versus *pK*_m _relationship (Figure 4). It was shown that recognition of *N*^β^-methylated substrates occurred on both binding and catalytic steps, while peptides and their aza-β^3^-derivatives were recognized primarily in their non-covalent binding step. This can be explained by the increase of backbone rigidity called forward by N^β^-methylation and the reduced ability of *N*^β^-methyl-aza-β^3 ^peptidomimetics to adopt conformations favorable for the phosphate transfer step in the protein binding site. In other words, N-methylation changed the orientation of the side-chains, thus hampering the substrates recognition by the protein. This conclusion was supported by the structure calculations of peptidomimetic substrates, which showed the preferred *cis*-configuration of -CO-NMe- peptide bond in three out of four *N*^β^-Me-aza-β^3^-amino acid-containing RRASVA derivatives, except *N*^β^-Me-aza-β^3^-RRASVA, where the methylated N-terminal amino group did not participated in peptide bond formation. This means that N^β^-methylation can be used as an efficient tool for tuning both peptidomimetics reactivity and selectivity for the target site, while for the non-methylated compounds only reactivity seems to be mostly affected.

## Methods

*N*^β^-Fmoc-*N*^β^-Me-aza-β^3^-amino acids were synthesized as described in [8]. Peptide analogs were prepared by solid-phase peptide synthesis using Fmoc/tBu methodology. *N*^β^-aza-β^3^-amino acid analogs were coupled as reported previously [7], using TBTU/HOBT as activators. For coupling of the amino acid following the *N*^β^-aza-β^3 ^residue, stronger activation was required and thus HATU/HOBT was used as activators.

The method of kinetic measurements was described in our previous article [5] and was based on utilizing the radioactive ATP with [^32^P]phosphate in γ-position. The phosphorylated substrates were bound onto Whatman phosphocellulose paper and the paper-bound radioactivity was counted. Linear plots between filter-bound radioactivity and time were used for calculating the initial velocity values of the phosphorylation reaction, which were thereafter processed by the classical Michaelis-Menten rate equation. Kinetic experiments were made at constant ATP concentration (100 μM) and the *K*_m _and *k*_cat _values were calculated for peptidomimetic substrates, concentrations of which were varied in the reaction mixture.

Peptidomimetic structure modeling was made using the Spartan 4.0 software suite (Wavefunction, Inc., USA) and the minimum energy conformations of compounds were obtained. Conformational searches were made using molecular mechanics with the additional condition of the aqueous medium for finding optimal geometry. All compounds were represented as zwitterions for these calculations.

## Abbreviations

HATU: 2-(1H-7-Azabenzotriazol-1-yl)-1,1,3,3-tetramethyl uronium hexafluorophosphate; HOBT: 1-hydroxybenzotriazole; TBTU: *O*-(Benzotriazol-1-yl)-*N, N, N',N'*-tetramethyluronium tetrafluoroborate.

## Competing interests

The authors declare that they have no competing interests.
